# TRPV4 channel activation selectively inhibits tumor endothelial cell proliferation

**DOI:** 10.1038/srep14257

**Published:** 2015-09-21

**Authors:** Roslin J. Thoppil, Ravi K. Adapala, Holly C. Cappelli, Vinay Kondeti, Andrew C. Dudley, J. Gary Meszaros, Sailaja Paruchuri, Charles K. Thodeti

**Affiliations:** 1 Department of Integrative Medical Sciences, Northeast Ohio Medical University, Rootstown, OH 44272; 2 School of Biomedical Sciences, Kent State University, Kent, OH 44240; 3 Department of Chemistry, University of Akron, Akron, OH 44325; 4 Department of Cell and Molecular Physiology, University of North Carolina, Chapel Hill, NC 27599.

## Abstract

Endothelial cell proliferation is a critical event during angiogenesis, regulated by both soluble factors and mechanical forces. Although the proliferation of tumor cells is studied extensively, little is known about the proliferation of tumor endothelial cells (TEC) and its contribution to tumor angiogenesis. We have recently shown that reduced expression of the mechanosensitive ion channel TRPV4 in TEC causes aberrant mechanosensitivity that result in abnormal angiogenesis. Here, we show that TEC display increased proliferation compared to normal endothelial cells (NEC). Further, we found that TEC exhibit high basal ERK1/2 phosphorylation and increased expression of proliferative genes important in the G1/S phase of the cell cycle. Importantly, pharmacological activation of TRPV4, with a small molecular activator GSK1016790A (GSK), significantly inhibited TEC proliferation, but had no effect on the proliferation of NEC or the tumor cells (epithelial) themselves. This reduction in TEC proliferation by TRPV4 activation was correlated with a decrease in high basal ERK1/2 phosphorylation. Finally, using a syngeneic tumor model revealed that TRPV4 activation, with GSK, significantly reduced endothelial cell proliferation *in vivo*. Our findings suggest that TRPV4 channels regulate tumor angiogenesis by selectively inhibiting tumor endothelial cell proliferation.

Endothelial cell (EC) proliferation is majorly regulated by soluble factors, such as vascular endothelial growth factor (VEGF), which is required for angiogenesis. However, mechanical forces, generated by pulsatile blood flow (shear and strain), also control EC proliferation, possibly via modulation of EC responses to soluble growth factors^1^,^2^,^3^. Matrix rigidity has been shown to influence cellular behavior, and cells grown on rigid matrices (such as within a tumor), as opposed to softer matrices, can trigger proliferative and survival pathways through alterations in gene expression^4^,^5^,^6^. Previous studies demonstrated that EC isolated from different tumors exhibit structural and functional characteristics atypical of the normal endothelium^7^,^8^. In fact, tumor endothelial cells (TEC), which make up the tumor vasculature, fail to sense mechanical cues accurately, resulting in malformed blood vessels^9^. These findings suggest that TEC could be a potential specific target for anti-angiogenic therapy.

While calcium has not been widely pursued as a potential anti-angiogenic target, intracellular calcium has been demonstrated to regulate angiogenesis, including the processes of cell proliferation, survival, and migration^10^,^11^. In fact, VEGF has been shown to stimulate Ca^2+^ influx in EC through ion channels, including members of the transient receptor potential (TRP) family^12^,^13^. We have previously confirmed that TRPV4 channels are activated by cyclic strain and mediate EC reorientation via activation of integrin to integrin signaling^14^. Additionally, we demonstrated TEC to exhibit aberrant mechanosensitivity, high ERK1/2 activity, and abnormal angiogenesis *in vitro*. However, the signaling mechanism(s) upstream of this abnormal TEC phenotype are not yet known. We recently found that TEC express reduced levels of TRPV4 compared to normal endothelial cells (NEC), and exhibit abnormal Rho-dependent mechanosensing and angiogenesis, which were restored upon overexpression or pharmacological activation of TRPV4^15^. However, it remains unclear if TRPV4 modulates TEC proliferation, a key event in angiogenesis.

In the present study, we investigated the role of TRPV4 in TEC proliferation, including the potential underlying molecular mechanism(s).

## Results

### TEC exhibit increased proliferation which is reduced by pharmacological activation of TRPV4

**Figure 1:**
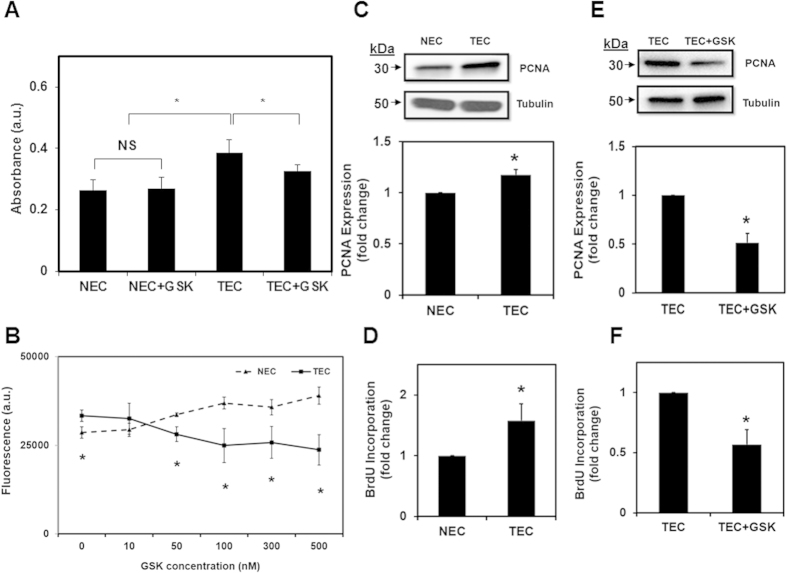
TEC exhibit increased proliferation which is inhibited by pharmacological activation of TRPV4. (**A**) XTT assay showing increased proliferation of tumor endothelial cells (TEC), compared to normal endothelial cells (NEC), which was significantly (p ≤ 0.05) reduced by GSK (100 nM). NS = non-significant. The data shown is mean ± SEM from five independent experiments. Significance was determined using Student’s paired *t* test and significance was set at p ≤ 0.05. (**B**) Concentration-dependent reduction in TEC proliferation by GSK measured using Calcein-AM (p ≤ 0.05). Note that GSK did not affect NEC proliferation. Significance was determined using Student’s paired *t* test and ANOVA with Tukey’s post-hoc analysis and significance was set at p ≤ 0.05. (**C**) Western blot analysis of PCNA expression in NEC and TEC. Quantitative analysis of the Western blots showing a significant (p ≤ 0.05) increase in PCNA expression in TEC. PCNA levels were normalized to tubulin and expressed as a fold change compared to NEC. (**D**) Quantitative analysis of BrdU positive cells in NEC and TEC (percentage of BrdU positive cells from the total number of cells (1000 cells for condition)) and expressed as fold change relative to NEC (p ≤ 0.05). (**E**) Western blot analysis of PCNA expression in TEC untreated or treated with GSK. Quantitative analysis of the Western blots showing a significant (p ≤ 0.05) decrease in PCNA expression in TEC treated with GSK. PCNA levels were normalized to tubulin and expressed as a fold change relative to TEC). (**F**) Quantitative analysis of BrdU positive cells in TEC untreated or treated with GSK (percentage of BrdU positive cells from the total number of cells (1000 cells for condition)) and expressed as fold change compared to TEC (p ≤ 0.05). All the data shown is mean ± SEM from at least three independent experiments.

We investigated if TEC proliferation levels are altered compared to NEC, which may contribute to the abnormal angiogenesis and structure of tumor vessels. We found that TEC exhibited significantly (p ≤ 0.05) increased proliferation compared to NEC, as measured by XTT and cell viability assays (Fig. 1A,B). However, GSK did not affect NEC proliferation (Fig. 1A,B). Further, Western blot analysis revealed that expression of proliferating cell nuclear antigen (PCNA) is increased in TEC compared to NEC (p ≤ 0.05; Fig. 1C). To further confirm that TEC exhibit increased proliferation, we measured incorporation of bromodeoxyuridine (BrdU) into the cells. Indeed, we found that TEC had significantly (p ≤ 0.05) higher numbers of BrdU positive cells (Fig. 1D). Morphologically, TEC are bigger than NEC, so to avoid any bias in counting the percentage of BrdU positive cells, we measured BrdU incorporation in 1000 cells (n = 1000 cells for each cell type).

Since TEC express low levels of TRPV4 (Supplemental Fig. S1;^15^) and pharmacological activation or overexpression of TRPV4 normalizes TEC mechanosensitivity and angiogenesis^15^, we investigated if targeting TRPV4 normalizes the increased TEC proliferation. We found that TRPV4 activation, using a small molecule activator GSK (100 nM), markedly reduced TEC proliferation (Fig. 1A,B), PCNA expression (Fig. 1E) and BrdU incorporation (Fig. 1F). In contrast, GSK treatment did not inhibit proliferation of NEC (Fig. 1A,B).

**Figure 2:**
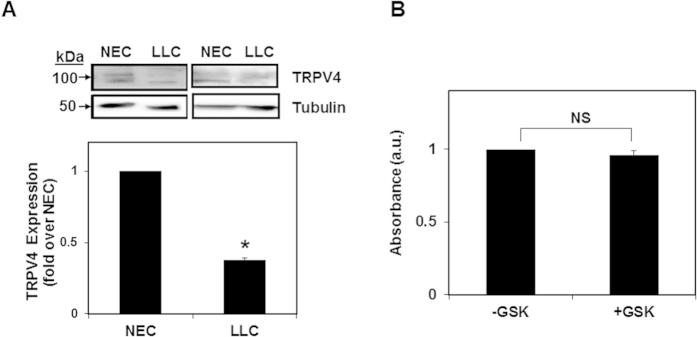
TRPV4 pharmacological activation did not inhibit LLC cell proliferation. (**A**) Western blots depicting TRPV4 expression in Lewis Lung Carcinoma (LLC) cells. NEC were used as a positive control for TRPV4 expression. (**B**) XTT assay showing no change in LLC proliferation by GSK (100 nM). NS = non-significant. Data shown are ± SEM from four independent experiments.

To further confirm the specificity of GSK on TEC proliferation, we investigated if GSK treatment inhibits Lewis Lung Carcinoma (LLC) cell proliferation. While LLC cells express TRPV4 (Fig. 2A; NEC were used as a positive control), treatment with TRPV4 activator GSK did not affect proliferation, confirming that GSK specifically inhibits proliferation of TEC, but not tumor epithelial cells (Fig. 2B). Overall, the above results demonstrate that TEC proliferate at a higher rate than NEC, and that TRPV4 activation by GSK decreases proliferation of TEC.

### TRPV4 regulates TEC proliferation via modulation of ERK1/2 but not AKT

**Figure 3:**
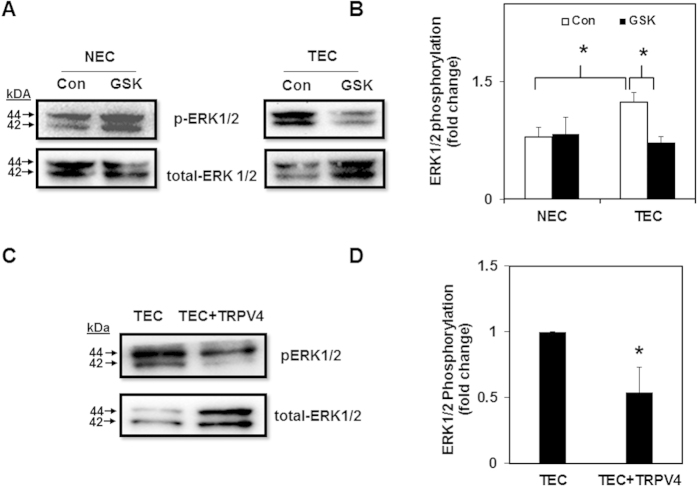
Pharmacological activation of TRPV4 decreases TEC proliferation via modulation of ERK1/2 but not AKT pathway. (**A**) Representative Western blots showing ERK1/2 phosphorylation in control and GSK treated NEC and TEC. (**B**) Densitometric analysis of the Western blots showing a decrease in ERK1/2 phosphorylation in TEC treated with GSK (100 nM). ERK1/2 phosphorylation was measured by normalizing phospho-ERK1/2 to total-ERK1/2 and was expressed as a fold change relative to NEC. (**C**) Representative Western blots depicting ERK1/2 phosphorylation TEC transfected with TRPV4-EGFP. (**D**) Densitometric analysis of the Western blots for ERK1/2 phosphorylation. ERK1/2 phosphorylation was measured by normalizing phospho-ERK1/2 to total-ERK1/2 and was expressed as a fold change relative to TEC. All the data shown is mean ± SEM from at least three independent experiments.

We next investigated the molecular mechanism(s) by which TRPV4 mediates the above effects. Since members of the mitogen-activated protein kinase (MAPK/ERK) family are critical regulators of cell proliferation, and mechanical signaling is known to stimulate ERK1/2^16^,^17^,^18^,^19^, we asked if TRPV4 regulates TEC proliferation via modulation of the ERK1/2 pathway. We found significantly (p ≤ 0.05) higher basal phosphorylation of ERK1/2 (p-ERK1/2) in TEC compared to NEC (Fig. 3A). Importantly, this up-regulation of ERK1/2 phosphorylation was inhibited when TEC were treated with GSK (100 nM), which remained unchanged in NEC (Fig. 3A,B). Further, overexpression of TRPV4 (Supplemental Fig. 2) also reduced high basal ERK1/2 phosphorylation in TEC (Fig. 3C, D). We next examined AKT phosphorylation in TEC, as many tumor cells exhibit increased survival rates and evade apoptosis, possibly through mechanisms that upregulate the PI3K-AKT pathway^20^,^21^. We found that basal AKT phosphorylation (p-AKT at Ser-473) was lower in TEC than NEC (Supplementary Fig. 3). Subsequently, we measured AKT phosphorylation in response to TRPV4 activation and found that GSK treatment markedly reduced p-AKT levels in NEC but only slightly increased in TEC (Supplementary Fig. 3). These findings suggest that TRPV4 activation or overexpression may reduce TEC proliferation via modulation of ERK1/2, but not the AKT pathway.

### Increased TEC proliferation is associated with enhanced expression of cell cycle genes

**Figure 4:**
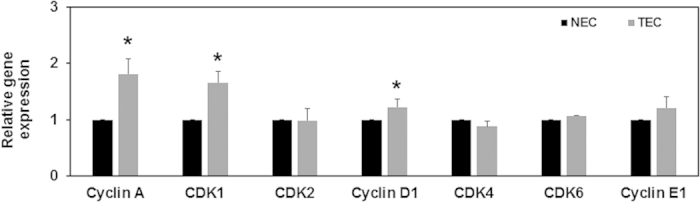
TEC express increased levels of proliferation-associated genes. Relative gene expression of cell-cycle associated genes in NEC and TEC. EC were treated with GSK (100 nM) for 24 h and subsequently lysed for RNA isolation. cDNA was prepared and qPCR analysis was performed using Fast SYBR green master mix (Applied Biosystems). Gene expression was first normalized to GAPDH and presented as relative expression to NEC. All the data shown is mean ± SEM from at least three independent experiments.

Activation of the ERK signaling pathway, as well as the extracellular matrix (ECM)/integrin/receptor tyrosine kinase signaling pathway, has been demonstrated to induce cyclin D1 mRNA expression, allowing entry of cells into S-phase^22^. We therefore measured gene expression of several cell cycle regulators (G1 and S phase) to determine if increased ERK1/2 phosphorylation in TEC, due to decreased TRPV4 expression, may induce up-regulation of these genes. Our results revealed that, when compared to NEC, there was a significant (p ≤ 0.05) increase in mRNA expression of several G1-S phase genes including cyclins A, D1, E1 and cyclin-dependent kinases (CDK) 1 and 6 (Fig. 4). These results suggest that TRPV4 deficiency in TEC may result in increased cell proliferation via ERK1/2 dependent modulation of cell cycle genes.

### Pharmacological activation of TRPV4 attenuated EC proliferation in tumors *in vivo*

**Figure 5:**
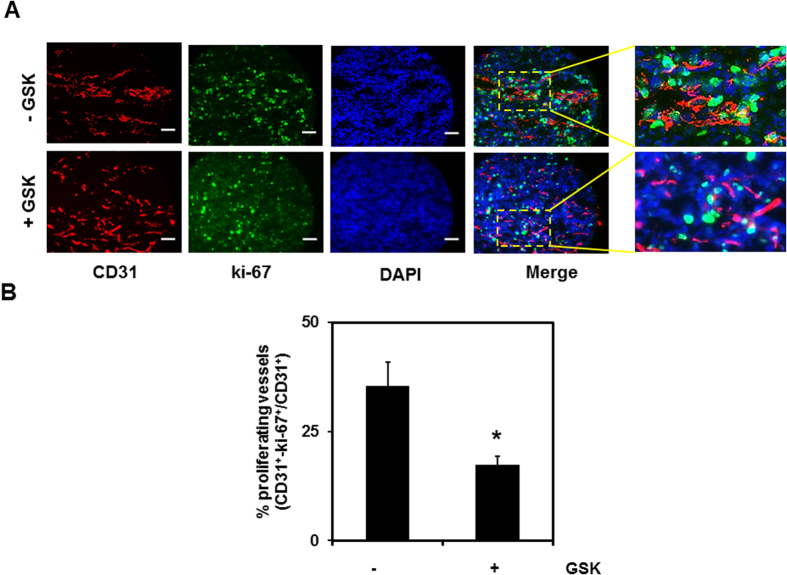
Pharmacological activation of TRPV4 inhibits EC proliferation *in vivo*. (**A**) Tumors were implanted into C57BL/6 mice by subcutaneously injecting LLC cells. Mice were treated with GSK (+GSK) or saline (−GSK), as previously described^15^, and isolated on day 21. Representative images (20X) of the tumor tissue stained with CD31 (red), ki-67 (green), and DAPI (nuclei) were used to quantify the proliferation of TEC. Scale bar = 10 μm (**B**) Quantitative analysis demonstrating a significant (p ≤ 0.05) decrease in the percentage of proliferating vessels (CD31+ki-67 positive vessels divided by CD31 positive vessels) in tumors treated with GSK (+GSK) compared to untreated tumors (−GSK).

Finally, to determine the functional significance of TRPV4 in the regulation of TEC proliferation *in vivo*, we induced LLC tumors in wild-type (WT) mice, as previously described^15^. We found that treatment with GSK, together with anticancer drug Cisplatin, significantly inhibited tumor growth, suggesting that activation of TRPV4 normalizes tumor angiogenesis and improves delivery of chemotherapeutic drugs. However, GSK treatment alone did not inhibit tumor growth (953.19 ± 290.11(untreated) vs 1174.84 ± 206.51(treated with GSK)^15^), further confirming that GSK has no effect on tumor cell growth. To investigate if GSK treatment regulates TEC proliferation, frozen sections of tumors, harvested from mice injected with saline (−GSK) or GSK (+GSK), were immunostained with the specific EC marker CD31 (red), the proliferation marker ki-67 (green), and nuclei (DAPI: blue). Proliferating EC in tumor vessels were identified by visualizing the co-localization of the CD31, ki-67, and DAPI (Fig. 5A). We found substantial levels of proliferating endothelial cells (both CD31/ki67 positive) in untreated (−GSK) tumors, which were markedly reduced in tumors that were treated with the specific TRPV4 activator, GSK (Fig. 5A,B). Quantitative analysis revealed a significant (p ≤ 0.05) reduction in the number of proliferating EC in tumors from GSK-treated mice (+GSK) compared to tumors from saline injected mice (−GSK) (Fig. 5B). Thus, the above findings provide evidence that TRPV4 regulates EC proliferation during tumor angiogenesis.

## Discussion

In the present study, we demonstrate that reduced expression of the mechanosensitive ion channel TRPV4 in TEC results in increased proliferation, which may contribute to abnormal tumor angiogenesis. We show that the pharmacological activation of TRPV4, with a small molecule activator GSK, decreases TEC proliferation *in vitro*. Further, we found a correlation between TRPV4 expression, abnormal TEC proliferation, and increased ERK1/2 phosphorylation, which was attenuated by pharmacological activation or overexpression of TRPV4. Finally, we demonstrate that EC proliferation in tumors is significantly inhibited by treatment with GSK in mice subcutaneously implanted with LLC tumors.

Several studies have indicated different TRP channels to influence the angiogenic process through multiple mechanisms^12^,^23^,^24^. However, there have not been any studies on TRP channels involved in tumor-derived endothelial cell (TEC) growth and function and their relevance to tumor angiogenesis. Recently, we demonstrated, for the first time, mechanosensitive TRPV4 channels regulate tumor angiogenesis by modulating Rho-dependent EC mechanosensitivity. Specifically, we have shown that the pharmacological activation of TRPV4 with GSK normalizes the tumor vasculature and, in combination with Cisplatin, reduces tumor growth in wild-type (WT) mice^15^.

Previously, TRPV4 dysfunction has been found to increase renal cystogenesis, and pharmacological activation of TRPV4 reduces proliferation of renal duct cells as well as cholangiocytes^25^,^26^. However, the role of TRPV4 in TEC proliferation had not yet been explored. The present study clearly demonstrates that TRPV4-deficient TEC display increased proliferation, which is normalized by pharmacological activation of TRPV4, suggesting that TRPV4 function is required for normal proliferation, and de-regulation of TRPV4 signaling may lead to abnormal proliferation. Further, our results demonstrate that TRPV4 regulates TEC proliferation via modulation of ERK1/2 phosphorylation. The activation of the ERK pathway is usually associated with the induction of cyclin D1 mRNA expression. Interestingly, it was shown that ERK activation, integrin signaling, cell cycle progression, and ultimately cell proliferation, relies on ‘tension-dependent changes in cell shape and cytoskeletal structure’^27^. We have previously shown that TRPV4 is a mechanosensor that is important for EC spreading, cytoskeletal remodeling, and reorientation^14^,^15^ and have recently shown that TEC exhibit decreased expression and function of TRPV4 channels. As a result, this causes abnormal mechanosensing in TEC, which exhibits uncontrolled spreading on ECM substrates with increasing stiffness (98–2280 Pa)^15^. These findings indicate that TRPV4-deficiency induces abnormal mechanosensitivity in TEC and may allow for increased spreading, cell cycle progression, and proliferation.

Indeed, our results have shown that TEC exhibit increased expression of cell cycle genes, S phase protein, PCNA, and proliferation. Importantly, in untreated animals (−GSK), we found increased co-localization of the proliferative marker ki-67 and endothelial marker CD31, confirming that there is increased proliferation of TEC in tumors. However, pharmacological activation of TRPV4 with GSK normalized ERK1/2 phosphorylation, TEC proliferation *in vitro,* and significantly reduced TEC proliferation *in vivo,* revealing a correlation between TRPV4-dependent ERK1/2 activity and TEC proliferation. We have previously shown that TRPV4 expression is lower in TEC compared to NEC and treatment with TRPV4 agonist, GSK, up-regulates TRPV4 expression in TEC but not in NEC. Further, GSK treatment normalized abnormal angiogenesis exhibited by TEC *in vitro*. Similarly, increased expression of TRPV4 by GSK may inhibit TEC proliferation via inhibition of ERK1/2 phosphorylation. Based on these findings, we hypothesize that TRPV4 expression (and/or activity) should be at a threshold level to maintain EC mechanosensitivity and proliferation, and beyond this threshold TRPV4 expression may not have any additional effects, as seen in NEC.

Taken together, our data supports a significant role for the mechanosensitive TRPV4 channel in regulating TEC proliferation via modulation of the ERK pathway. We have demonstrated that pharmacological activation of TRPV4 induces vascular normalization in tumors by modulating Rho activity^15^ and may improve chemotherapeutic drug delivery, which consequently reduced tumor growth. Our current study presents an additional role for the mechanosensitive ion channel TRPV4 in the proliferation of TEC, a key event during tumor angiogenesis. Understanding the functional significance and molecular signaling behind the TRPV4-dependent regulation of TEC proliferation and tumor angiogenesis could provide a novel avenue for anti-angiogenic or vascular normalization therapies.

## Methods

### Cell culture

Normal (NEC) and tumor EC (TEC) were extensively characterized and cultured on fibronectin or gelatin coated tissue culture dishes and grown in a defined medium in a 37 °C, 5%CO_2_ incubator, split at ~90–95% confluence, and used between passages 11–22, as previously described^9^,^28^. Mouse Lewis Lung Carcinoma (LLC) cells were cultured in high glucose DMEM medium supplemented with 10% FBS and antibiotic/mycotic mix at 37 °C.

### **SDS-PAGE and Western blot Analysis**

Cells were treated with (GSK: GSK1016790A 100 nM) or vehicle (DMSO), washed with PBS, and lysed in RIPA buffer or Triton X-100 with protease and phosphatase inhibitors (Boston Bioproducts). Lysates were loaded and separated by electrophoresis on 8%, 10%, or 12% SDS acrylamide gels. Proteins were transferred onto a PVDF membrane and the membrane was blocked in 5% milk in TBS with 0.01% Tween-20 (TBS-T) for 1 h. The blots were incubated with primary antibodies: phospho-ERK1/2 (1:1000) (Cell Signaling); total ERK1/2 (1:1000) (Cell Signaling); phospho AKT (1:1000) (Cell Signaling), total AKT (1:1000) (Cell Signaling), anti-PCNA (1:1000) (Abcam) and tubulin (1:5000) (Abcam) overnight, rinsed three times with TBS-T, and incubated with the appropriate secondary antibody goat anti-mouse (1:20,000) or goat anti-rabbit (1:20,000) conjugated with horseradish peroxidase (Jackson Laboratories). Signals were detected using chemiluminescent substrates (Thermo Scientific) and developed with a FluorChem M Simple Imager (Protein Simple).

### qPCR

RNA isolation of cells was performed using an RNeasy Mini Kit (Qiagen) and measured using the NanoDrop 2000 UV-Vis Spectrophotometer. cDNA synthesis was performed using qscript cDNA SuperMix (Quanta Biosciences) and qPCR analysis was performed using the Fast SYBR green master mix (Applied Biosystems) on the Fast Real-Time PCR system (Applied Biosystems). Real time PCR was performed using real time cyclin D1, cyclin A, cyclin E1, CDK1, CDK2, CDK4, CDK6, TRPV4, and GAPDH primers obtained from IDT technologies. Gene expression was first made relative to GAPDH and ΔΔCT values were expressed as fold change compared to NEC.

### Transfection

Cells were transfected with TRPV4-EGFP or EGFP constructs using targefect (Targeting Systems)^15^,^29^. The transfection efficiency was found to be 80–90%^15^,^29^. The expression of TRPV4- EGFP in EC was visualized using a Nikon Eclipse TE 2000-E microscope (Nikon, Japan) fitted with a CoolSnap HQ digital camera (Photometrics) or Olympus IX72 fluorescence microscope (Olympus).

### Tumor model and immunohistochemistry

All animal experiments were performed according to an approved protocol by Northeast Ohio Medical University, IACUC. Mouse Lewis Lung Carcinoma (LLC) cells (2 × 10^6^) were subcutaneously injected in the flank region of C57BL/6 mice. Once the tumors were palpable (after 7 days), mice either received daily intraperitoneal (i.p.) injections of TRPV4 agonist GSK1016790A (10 μg/kg) (+GSK) or saline (−GSK), until day 21, as previously described^15^. On day 21, mice were euthanized and tumor tissues were collected and frozen in OCT (Tissue Tek). *In vivo* proliferation assays were performed using frozen sections (10 μm; collected from both the central and peripheral regions of the tumor and at least 9 sections from each condition) fixed and permeabilized in ice cold acetone (20 min), washed with Tris buffered saline (TBS), and incubated with rat-anti-CD31 (Invitrogen) (1:50) and anti-ki-67 (Abcam) (1:200) overnight. Sections were then washed with TBS (3x–5 min) and incubated for 1 h with appropriate secondary antibodies coupled to Alexa Fluor-488 or Alexa Fluor-594 (Invitrogen) and mounted with DAPI (Vector labs). Images were acquired with Olympus Epifluorescence Microscope (IX71) using QCapture Pro (QImaging) and quantified using ImageJ. Total number of endothelial cells (CD31^+^ stained vessels) as well as the number of EC that co-localized with ki-67 were counted and expressed them as % of proliferating EC (CD31^+^-ki-67^+^/CD31^+^). The rationale for this is to differentiate proliferating EC (vessels) from non-proliferating EC.

### BrdU proliferation assay

Cells were counted and plated equally at low density on cover glasses and cultured for 24 h. Cells were treated with 10 μM BrdU (Abcam) for 2 h. The cells were fixed with 4% PFA, washed with PBS containing 0.1% Triton X-100 (PBST) (3x–5 min), and incubated for 10 min with 1N HCL on ice. Fixed cells were then incubated with 2N HCL at room temperature (RT) for 10 min and moved to the incubator (37 °C) for 20 min. Borate buffer (0.1 M) was used to buffer the cells for 12 min at RT. Cells were washed with PBST (3x–5 min), blocked with PBST solution containing 1M glycine and 5% fetal bovine serum (FBS) for 1 h, and incubated with anti-BrdU primary antibody (1:100) (Abcam) overnight at RT. Following incubation, cells were washed with PBST (3x−5 min), incubated with secondary antibody, Alexa Fluor-488 (Invitrogen), and mounted with DAPI (Vector labs). Images were captured using an Olympus IX71-fluorescence microscope, and analyzed with ImageJ. Quantitative analysis was performed by measuring BrdU positive cells in NEC and TEC (percentage of BrdU positive cells from total 1000 for condition) and expressed as fold change over respective control conditions (NEC in the case of NEC vs TEC or TEC in the case of TEC vs TEC+GSK) (p ≤ 0.05).

### Cell proliferation assay

Cell proliferation was determined using XTT kit (Biotium) and cell viability kit (Enzo biosciences). Cultured EC were trypsinized, counted, and plated in a 96-well plate (1000–2000 cells/well). 24–48 h post plating, media was removed and cells were treated with GSK1016790A (GSK) (10–500 nM) in serum free media for overnight. Cells were washed once with PBS; and XTT or Calcein dye was added to the cells and incubated for 30 min-24 h. Wells containing no cells with the dye served as controls to determine the level of the background signal. Absorbance was read at 485–535 nm or 450–500 nm, respectively for Calcein and XTT.

### Statistical analysis

All the data shown is mean ± SEM from at least three independent experiments. Significance was determined using Student’s *t* test, analysis of variance (ANOVA) and Tukey’s post-hoc analysis with significance set at p ≤ 0.05.

## Additional Information

**How to cite this article**: Thoppil, R. J. *et al.* TRPV4 channel activation selectively inhibits tumor endothelial cell proliferation. *Sci. Rep.* **5**, 14257; doi: 10.1038/srep14257 (2015).

## Supplementary Material

Supplementary Information
