# Supplementary material for: TRPV4 channel activation selectively inhibits tumor endothelial cell proliferation
Source: Sci Rep. 2015 Sep 21;5:14257. doi: 10.1038/srep14257 (PMC4585691; doi:10.1038/srep14257)
Supplement: Supplementary Information [file srep14257-s1.pdf]

TRPV4 channel activation selectively inhibits tumor endothelial cell proliferation

Roslin J. Thoppil[1,2#], Ravi K. Adapala[1,2#], Holly C. Cappelli[1,2], Vinay Kondeti[3], Andrew C. Dudley[4], J. Gary Meszaros[1,2], Sailaja Paruchuri[3], and Charles K. Thodeti[1,2]*

[1]Department of Integrative Medical Sciences, Northeast Ohio Medical University, Rootstown, OH 44272; [2]School of Biomedical Sciences, Kent State University, Kent, OH 44240; [3]Department of Chemistry, University of Akron, Akron, OH 44325; [4]Department of Cell and Molecular Physiology, University of North Carolina, Chapel Hill, NC 27599.

# These authors contributed equally to this work.

Running Title: TRPV4 regulates tumor endothelial cell proliferation.

*Address all correspondence to: Charles K. Thodeti, Ph.D., Department of Integrative Medical Sciences, Northeast Ohio Medical University, Rootstown, OH 44272 (Tel: 330-325-6423; Fax: 330-325-5912; Email: cthodeti@neomed.edu)

Keywords: Angiogenesis, Endothelial, ERK1/2, Proliferation, Tumor Endothelial Cell, TRPV4

Supplementary Information

Supplementary Figure Legends:

Fig.S1. qPCR analysis showing reduced expression of TRPV4 mRNA in TEC compared NEC.

Fig.S2. Photomicrographs showing the efficiency of TRPV4-EGFP transfection in tumor endothelial cells.

Fig.S3. A) Representative Western blots depicting AKT phosphorylation in NEC and TEC treated or untreated with GSK (100 nM). **B)** Densitometric analysis of the Western blots for AKT phosphorylation. AKT phophorylation was measured by normalizing phospho-AKT to total-AKT. Significance was set at $p \leq 0.05$ and compares NEC to NEC treated with GSK and NEC to TEC.

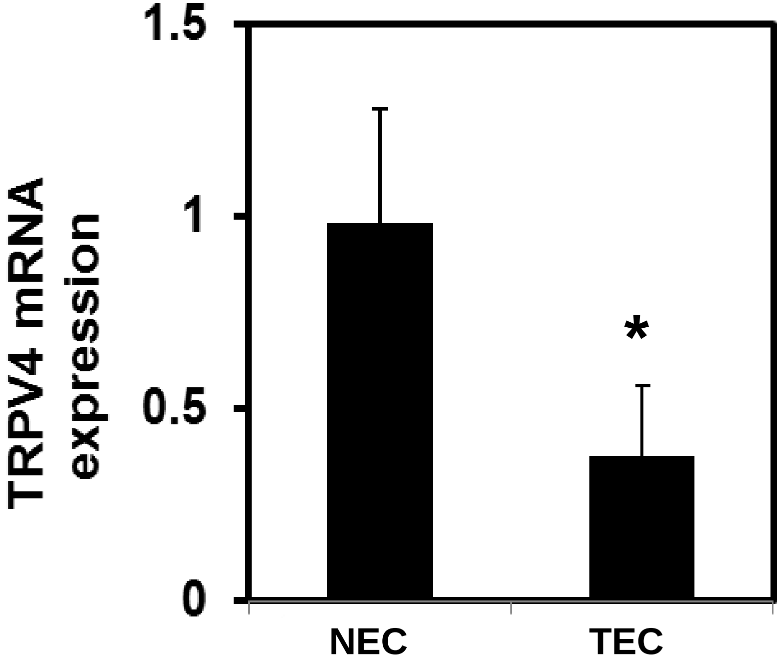
TRPV4 mRNA expression
1.5
1
0.5
0
*
NEC
TEC

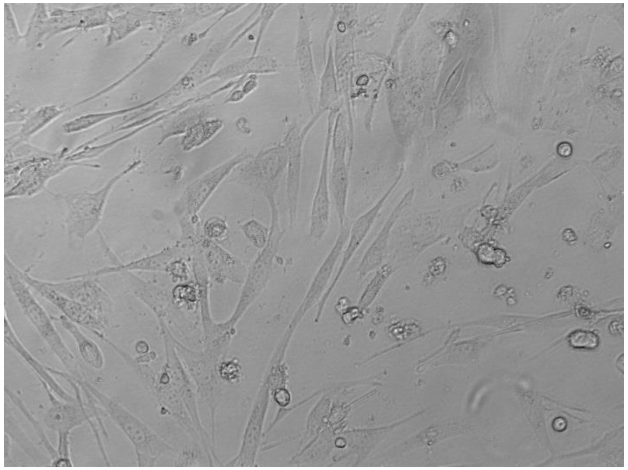

Phase contrast

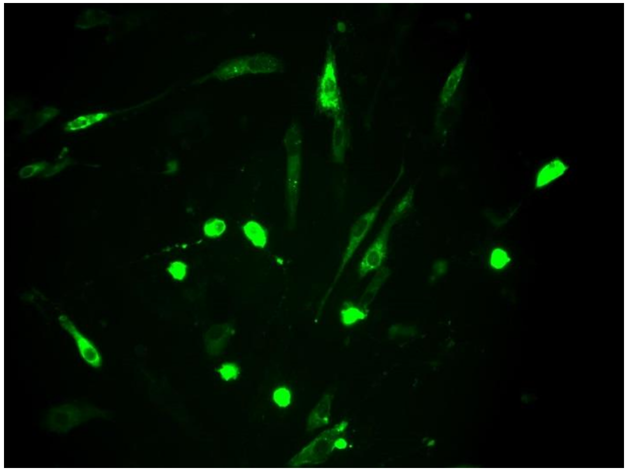

EGFP

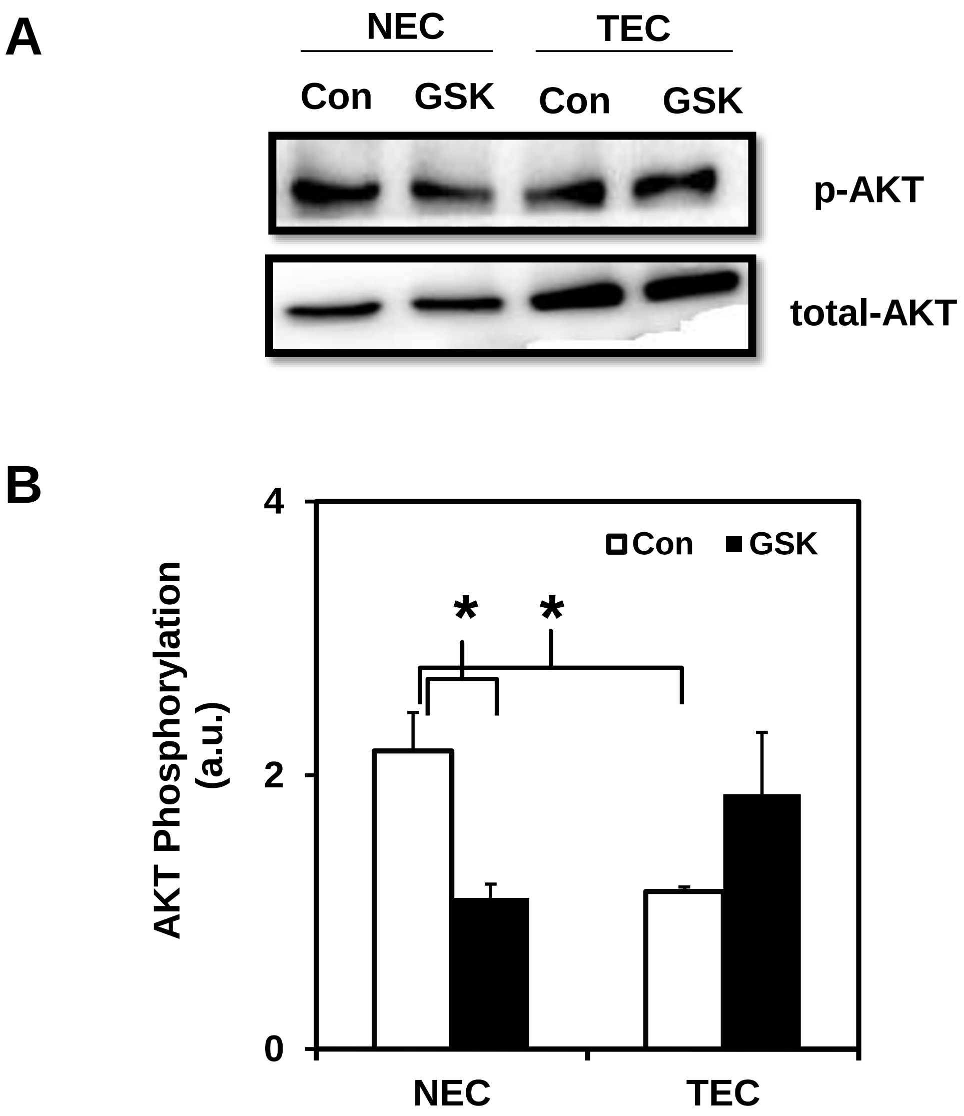
A
NEC
TEC
Con
GSK
Con
GSK
p-AKT
total-AKT
B
Con
GSK
*
*
AKT Phosphorylation (a.u.)
4
2
0
NEC
TEC
